# Supplementary material for: Quercetin Attenuates KLF4-Mediated Phenotypic Switch of VSMCs to Macrophage-like Cells in Atherosclerosis: A Critical Role for the JAK2/STAT3 Pathway
Source: Int J Mol Sci. 2024 Jul 15;25(14):7755. doi: 10.3390/ijms25147755 (PMC11277168; doi:10.3390/ijms25147755)
Supplement: Supplementary file 1 [file ijms-25-07755-s001.zip › Supplementary Figure S1.pdf]

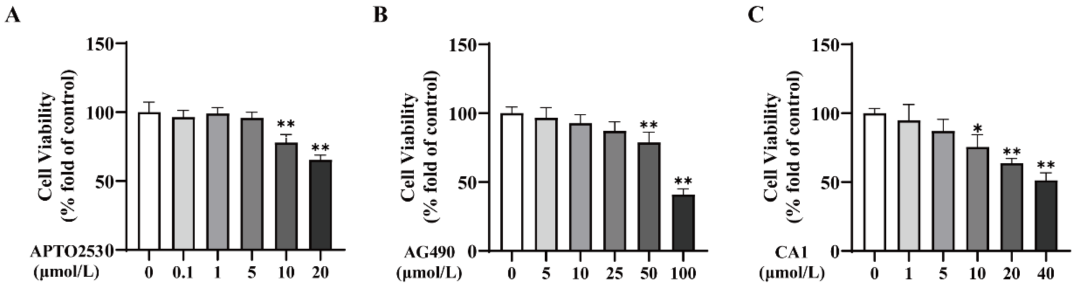

Figure S1. Evaluation of MOVAS cell viability upon exposure to various concentrations of APTO-253 (A), AG490 (B) and CA1 (C) using the CCK-8 assay.
